# What Is Critical About the Crisis of Expertise? A Review of Gil Eyal’s *The Crisis of Expertise* (2019, Cambridge: Polity Press)

**DOI:** 10.1007/s10767-021-09402-x

**Published:** 2021-05-17

**Authors:** Riccardo Emilio Chesta

**Affiliations:** Faculty of Political and Social Sciences, Scuola Normale Superiore, Florence, Tuscany Italy

## The Critical in the Crisis

THE LITTLE MONK: And do you not believe that the truth – if it be the truth will triumph even without us?

GALILEO: No, no, no. Truth will triumph only in so far as we triumph; the victory of reason can only be the victory of reasonable people.

(B. Brecht, *Life of Galileo,* Scene VIII)

Particularly in these pandemic times, appeals to the state of crisis as well as to the rethinking of expertise are omnipresent. Though published few months before COVID-19 disrupted ordinary frictions between politics and expertise, Gil Eyal’s *The Crisis of Expertise* (2019) helps us going directly into the *vortex* of this crisis, while at the same time avoiding any shortcut.

In contrast to sensational disquisitions regarding the death of expertise, claims about a “science under assault” and the spread of fake news, Eyal’s book is an intellectual contribution which tries to tidy up a long debate on expertise. Stuck in the hybrid nature of its public-scholarly aporias, this debate got further complicated by the public relevance of these controversies, characterized by conflicts of value or interests, and uncertainties about the legitimate procedures to solve them.

Despite these dramatic complications, Gil Eyal clearly moves away from purely normative or moralistic postures. In this essay, he gives a critical overview of key issues regarding the studies of expertise and offers important insights to interpret many of the most important experiments of technical democracy. He does it with a deep theoretical understanding, intellectual verve, and even a certain prophetic spirit characteristic of the sociology of science since Merton.

The result is an agile, but densely written book that clears some of the many misunderstandings of *that obscure object of desire* that takes the name of *expertise.* While re-defining many issues, *The Crisis of Expertise* poses new ones and begs the question: “if expertise is in a *crisis*, what is *critical* about expertise?”

Eyal suggests that the nature of this *crisis* needs to be traced in the *real* nature of expertise, defined as a *historically specific way of talking.* This is strictly related to two essential albeit slippery elements of social life: *trust* and *risk. Expertise*,* trust*, and *risk* represent a critical triad around which the book is organized and where the origins of the crisis are located. As such, they are key to the analysis of the many technocratic or democratic experiments designed to solve the complex controversies of our time. In the attempt to negotiate and obtain objectively shared principles, these experiments produced institutionalization processes or legal procedures that in most cases fetishized their implicit political philosophies into *science-policies*.

The large variety of strategies devised to get out of the *swirling vortex* of techno-scientific controversies is classified by Eyal in three/four categories: *exclusion*, or a “boundary-work” that confines controversy to technocratic expert judgment; *inclusion*, or the extension of controversy to the participation of lay people in order to improve transparency; *mechanical objectivity*, or the search for objective and standardized procedures that reduce human judgment and error; and *outsourcing*, or a strategy of expertise spin-off (a category that frequently intersects with the previous ones).

However, Eyal explains that these strategies, rather than solving controversies, they are *performative* tools that change controversies transforming how issues are defined and the way struggles are conducted:

The examples of tensions and contradictions can be multiplied further, but the point hopefully is clear: the responses to the legitimacy crisis backfire and exacerbate it, especially as they spar with one another. Science itself becomes infected, and the attempts to organize, pluralize, mechanize, or outsource expertise are all caught in a self-reinforcing vortex of mutual pollution and mutual undermining. Yet, albeit tension-ridden and crisis-prone, the entanglement of science and the state survives and even expands. However mistrusted or doubted, expertise is even more indispensable and essential than ever before (p.129).

While acknowledging the real and indispensable function of expertise, Eyal also follows Alvin Weinberg in defining the “trans-scientific” nature of most of the questions for which expertise is the answer.

“Expertise is our name for this realm of trans-science, where questions are asked that *should* be asked but *cannot* be answered” (p.144). Eyal’s main argument is thus partly rooted in Weberian’s theory of scientific practice as *Beruf* whose role is nothing more than helping the parties in conflict to recognize “inconvenient facts”. But here, the ambivalences of Weberian epistemology fully emerge.

No phenomenological *epoché* of pre-existing values and beliefs indeed can be made without a preexisting political subject that experiences the discovery of facts and recognize them as inconvenient, discomfiting, or, on the opposite, encouraging. Weber’s science as vocation cannot be read in this sense without its political counterpart (Weber, 1919).

## Legitimate Expertise in Democracy

Though expertise is defined as a *historically specific way of talking* about the *friction between science and technology on the one hand, and law and democratic politics on the other*, Eyal sees his approach as *realist* and distinguishes it from those discursive or narrative frameworks which reduce the role and impact of expertise in society: *some people have it in their minds and hands* (p. 20–21). Expertise is therefore regarded as a practical knowhow gained by individuals through experience and practice.

By defining expertise and its crisis, this is once again distinguished from “science” and any crisis of science. Science is a term used equivocally to define a myriad of scientific applications, network of specific knowledge and techniques which are at the core of innovation-driven societies. In contrast, *the science that matters* and which keeps going the many *vortexes* of controversies is the one in service of specific, and therefore conflicting, *interests*. This is the reason why in current public, disputes expertise and not science has gained a more and more relevant role.

The recent history of the many variants of representative democracies—or as Dahl would say, “polyarchies”—is also the history of the various experiments to domesticate conflict and reach a consensus around the institutional definition and regulation of expertise. To define *legitimate* expertise, make a distinction among the many available, or draw a line separating expert from charlatans, entails a search for shared principles and procedures. Because it concerns legitimacy, this process is necessarily political*.*

However, the risks and the uncertainty intrinsically produced by our innovation-driven democracies constantly expose these principles and procedures to constant negotiation and redefinition. Because these principles and procedures are always transitory, *crisis* should not be considered a pathological state of degradation or decadence, but intrinsic to their nature. Therefore, innovation is by definition linked to risk, and both are linked to uncertainty and complexity. The complexity of the socio-technical operations that shape innovation can be in fact defined by the uncertainties intrinsic to the *abstract* procedures that the main actors—scientists, technologists, stakeholders—select among many others. Contrary to the managerial dreams of Frederick Winslow Taylor and contemporary supporters of *mechanical objectivity*, there’s no *one-best way.*

Contemporary struggles on legitimate expertise are neither composed by a monolithic science or “scientific method,” nor by clearly defined disciplinary fields. They are instead constantly reworked by a collection of sub-disciplines (or better, networks of knowledge and techniques) called “policy science” or “regulatory science” whose ultimate goal is to produce policy proposals, legal frameworks, and regulations. Furthermore, such networks of knowledge and techniques deal more with the domestication of political conflict rather than with a scientific consensus which at first is rarely visible even to experts. At the same time, it can be said that the very existence of these networks is linked to a dialectical process (a Mertonian “self-fulfilling prophecy”): the more a political consensus is reached on their results, the more their practices become scientific.

Risk plays a key role in this story of crisis of expertise. Though controversial, this black box has become a successful dispositive engendering an ever-expanding multiplicity of research policy programs. Hegel’s image of the owl of Minerva well describes risk analysis: it takes his flight only in the twilight.

Eyal’s arguments against the existence of this collection of subdisciplines that goes under the name of “policy-sciences” are essentially four: first, turning upside down Beck’s adage “there’s no expert on risk” he argues that: “there’s no expert on risk because there are too many experts on risk” (p.64); second, no one has the relevant expertise to the problem at hand; and third, risk breaks boundaries between disciplines and professions, experts, and lay people; with the fourth “risk analysis is ethics and politics camouflaged by numbers” (p.74). Eyal definitely excludes the very scientific existence of risk expertise.

But are not these arguments that can be also applied to the never-ending debate on the definition of expertise and its boundaries? With regard to problems of society? One may ask how many types of expertise can be said to be predictive or easily replicable in time and space? Probably Eyal’s critique here is directed at the institutional spread of risk analysis research that tells us more about the political support behind their networks than about autonomy and scientific coherence.

But time matters not only for risk analysis. Because networks of expertise do not operate in a vacuum, *temporal constraints* are key to understand the outcomes of an expert-based democratic decision-making facing risk and innovation. To explain the temporal discrepancy between scientific research and political action, Eyal convincingly uses the metaphor of the *three-lane highway* (p.7): the left, fastest lane, is occupied by law and policy, while scientific research drives on the right, the slowest one; in the middle, where the two must adjust to one another, run both regulatory science and policy science.

Convincing as it looks, this description, however, does not seem to pay much attention to the role of media that, using Eyal’s metaphor, functions more as a car that in a hit-and-run disrupts the already precarious rules of the highway than as a police patrolling traffic. As Eyal admits, this sphere is an easy target for the “merchants of doubts” whose main objective is not manufacture of consent, but strategic dissent.

## Agnotology and the Scientization of Ignorance

Despite Robert K. Merton’s warnings against the *ambivalences* of men of science (1973), technocrats have willingly or not tended to propose the idea of expertise as a way to make things clearer and reduce complexity. On the contrary, an injection of more expert knowledge in a controversy by emphasizing uncertainty and relativism weakens the various arguments in favor of and against as well as the *credibility* and *reputation* of expertise itself. The acknowledgment that the art of exploiting these ambivalences is a “politics by other means” is thus not surprising (Latour, 1988).

If we go back to the quote from scene VIII of Brecht’s *Life of Galileo* (1938), we begin to realize that what has changed is not only the role of “expert knowledge” but of ignorance. In this famous scene, an imaginary dialogue set in Rome in 1616, the Little Monk argues strongly in favor of the utility of compassionate ignorance that keeps humble peasants in a state of hope, whose function is to make worldly sufferance acceptable despite all the misery and toil and maintain order. The monk sees free scientific inquiry as the disruptive force that threatens social order because it erases all the eschatological illusions borne out of despair and makes more bearable the tragic destiny of the poor. Here, ignorance is typically conceived as a performative tool for social order and as a justification against the subversive and liberatory potential of science.

In opposition to the Little Monk, Galilei embodies revolutionary reason; he is the founder and leader of a scientific movement for social progress and the partisan of a rational thinking that subverts any authority and hierarchy that oppress people by keeping them chained to superstition.

Both the monk and Galilei’s positions seem untenable if we look at the current dynamics of *scientization of politics* and *politicization of science* as well as to the strategies of “agnotology” discussed in Eyal’s book. In current technologically advanced societies, ignorance has become a much more sophisticated weapon. In some cases, it is used by powerholders to attack the credibility of scientific evidences tackling their interests, while in other cases, it weakens the disruptive power of corporate techno-scientific innovation; both the European institute of the *precautionary principle* and the European response to the GMOs controversy are well-known instances.

Furthermore, as Eyal correctly shows, the strong subversive virtues of science exposed in Brechtian pièce are nowadays used to build new and poorly justified hierarchies and authorities meant, on the one hand, to establish a technocratic regime and on the other to freeze democratic procedures under a collection of techniques and expertise “isolated from public scrutiny” (p.105).

Ignorance has many meanings and can be classified on many levels. Ignorance can be *ignorance about something* (we know what we do not know) or *ignorance about ignorance* (we do not know what/that we do not know). It can be “deliberate” ignorance when we decide not to take into account something depending on tradition, beliefs, interests, or simply natural because of our experiential limitations. Our human condition is made up of the total of experiences that we have as individuals living within the natural and social context to which we adapt while attempting to transform it. Thus, following Dewey, while these limits draw the blurred borders of our experience, our active will can expand it. Therefore, *ignorance that matters* is defined by the same components of our experience that tell us *which are the things that matters*. Ignorance that matters refers to a *part* of the experience we consider relevant and that we try to *appropriate* by overcoming it through *participation*. In this sense, participation cannot be reduced to a tool of the new *governamentality* with specific rules, a selected public, a temporal framework, the legitimate or even lucrative questions, and outcomes (Pellizzoni & Ylönen, 2012). It has to be conceived in its emancipatory potential—a mechanism that activates learning processes—and in its specific limits—it cannot be activated everywhere in the same way, and it cannot affect everybody in the same way. In this case, the experience of common citizens, like that of scientific experts, is specific and not affected in the same way by all the spheres of ignorance. Though not all-powerful, it is properly the function of intellectuals—and not experts—to recognize and point out the critical components, the trans-scientific nature of the issues at stake. Eyal at the end of his book resists the temptation to illustrate the conditions of possibility for *Beruf*.

## Extending the Republic of Trans-Science

Gil Eyal’s book is an important contribution to the field and at the same time a valuable guide for scholars (hopefully in various scientific departments), policy-makers, activists, and concerned citizens. The proposals for a republic of trans-science sketched in the concluding chapter are suggestive. While advocating for a renewal of the Weberian political lesson and the need of a “revalued, rededicated, professionalized and emboldened *civil service*” (p.149), he also explicitly proposes to institutionalize an “active, combative *irony*” where the recognition of inconvenient facts becomes a commonly shared practice. However, he does not say what the conditions that would make such exercise possible are.

In the end, the republic of trans-science is subject to the same recurrent dialectics of democracy. The only difference resides in the power of techno-scientific innovation that elevates them at a further level of technical abstraction and uncertainty. The nature of techno-scientific innovation has always been conflictual since it is a function of a broader political economy. While it has always been already difficult to perform a Weberian ethics of science in the so-called ivory tower, in social life, we are often obliged to admit our partisan nature. In Weber (1907), this was occasionally admitted in terms of speeches of a “class-conscious bourgeois.”

In the current Babylon of sub-disciplines, the tendency of expertise to transgress its own areas of competence has generated frictions/conflicts with moral and political issues strategically hidden by the means of the fetishized rhetoric of “mechanical objectivity” deployed to shun the responsibility intrinsic to politically relevant issues.

But as Sartre reminds us, dealing with things out of the specific concerns and going beyond the area of competence are typical characteristics of intellectuals (Sartre, 1972).

Eyal’s proposal for better institutions for the trans-scientific debate is welcome, so long as we are aware of the limits of the pluralist heaven, which sings with the upper-class accent of its heavenly chorus. For the same reason, we need not only to reinforce these institutions but to extend them. To navigate complexity, we need new collective intellectuals and new networks of expertise that are able to foster the rights of the unrepresented and the powerless and make public institutions work against those in position to impose force pure and simple over the force of the better argument. Institutions should provide the conditions of production for the practice of a reflexive expertise, able to recognize and criticize the rhetoric of techno-scientific determinism, conscious of the specificity of its conditions of production, its scientific contribution, and its public role of collective empowerment. Once again, it is necessary to rediscuss the conditions that allow the art of *parresia*.
